# Pretreatment with transforming growth factor beta-3 protects small intestinal stem cells against radiation damage in vivo.

**DOI:** 10.1038/bjc.1997.249

**Published:** 1997

**Authors:** C. S. Potten, D. Booth, J. D. Haley

**Affiliations:** Paterson Institute for Cancer Research, Christie Hospital NHS Trust, Manchester, UK.

## Abstract

The gastrointestinal tract, with its rapid cell replacement, is sensitive to cytotoxic damage and can be a site of dose-limiting toxicity in cancer therapy. Here, we have investigated the use of one growth modulator to manipulate the cell cycle status of gastrointestinal stem cells before cytotoxic exposure to minimize damage to this normal tissue. Transforming growth factor beta-3 (TGF-beta3), a known inhibitor of cell cycle progression through G1, was used to alter intestinal crypt stem cell sensitivity before 12-16 Gy of gamma irradiation, which was used as a model cytotoxic agent. Using a crypt microcolony assay as a measure of functional competence of gastrointestinal stem cells, it was shown that the administration of TGF-beta3 over a 24-h period before irradiation increased the number of surviving crypts by four- to six-fold. To test whether changes in crypt survival are reflected in the well-being of the animal, survival time analyses were performed. After 14.5 Gy of radiation, only 35% of the animals survived within a period of about 12 days, while prior treatment with TGF-beta3 provided significant protection against this early gastrointestinal animal death, with 95% of the treated animals surviving for greater than 30 days.


British Journal of Cancer (1997) 75(10), 1454–1459
© 1997 Cancer Research Campaign

### Pretreatment with transforming growth factor beta-3 protects small intestinal stem cells against radiation damage in vivo

**CS Potten**[1], **D Booth**[1] **and JD Haley**[2]

[1]Paterson Institute for Cancer Research, Christie Hospital NHS Trust, Wilmslow Road, Manchester M20 9BX, UK; [2]Oncogene Science, 106 Charles Lindbergh Blvd, Uniondale, New York 115533–3649, USA

**Summary** The gastrointestinal tract, with its rapid cell replacement, is sensitive to cytotoxic damage and can be a site of dose-limiting toxicity in cancer therapy. Here, we have investigated the use of one growth modulator to manipulate the cell cycle status of gastrointestinal stem cells before cytotoxic exposure to minimize damage to this normal tissue. Transforming growth factor beta-3 (TGF-β3), a known inhibitor of cell cycle progression through $G_1$, was used to alter intestinal crypt stem cell sensitivity before 12–16 Gy of gamma irradiation, which was used as a model cytotoxic agent. Using a crypt microcolony assay as a measure of functional competence of gastrointestinal stem cells, it was shown that the administration of TGF-β3 over a 24-h period before irradiation increased the number of surviving crypts by four- to six-fold. To test whether changes in crypt survival are reflected in the well-being of the animal, survival time analyses were performed. After 14.5 Gy of radiation, only 35% of the animals survived within a period of about 12 days, while prior treatment with TGF-β3 provided significant protection against this early gastrointestinal animal death, with 95% of the treated animals surviving for greater than 30 days.

**Keywords**: transforming growth factor beta; intestinal stem cells; cell cycle arrest; radiation protection; clonogenic cells

Damage to the stem cells of renewing tissues is a serious adverse side-effect of cancer therapy treatments. The moist stratified epithelia (oral and genital) and the simple columnar epithelia in the small bowel represent the most sensitive of these renewing tissues. Damage to the stem cells in these sites results in a cellular depletion which is manifested at times to be equivalent to the transit time through the tissue. In murine small bowel, this is equivalent to 3–4 days and in human oral mucosa to about 10 days.

We designed a series of experiments to investigate whether transforming growth factor beta-3 (TGF-β3) could be used to modulate the sensitivity of gastrointestinal stem cells. With the increasing use of haematopoietic cytokines to combat myelosuppression (Morstyn et al, 1994), gastrointestinal and oral mucositis are increasingly becoming dose- and schedule-limiting toxicities in standard- and high-dose chemotherapy regimens. Chemotherapy agents that cause oral or gastrointestinal mucositis include commonly prescribed agents, such as 5-fluorouracil (5-FU), doxorubicin, vinblastine, methotrexate, taxol and etoposide. The incidence of gastrointestinal mucositis is thought to be similar to that of oral mucositis, which occurs in approximately 20–25% of chemotherapy patients (Chabner, 1993).

The general principles of such experiments fall within the outlines given below. An appropriate delivery of factors to reversibly prevent cycle progression and cause an accumulation of cells in $G_1$ or $G_0$ before exposure to a cytotoxic agent may render the stem cells more resistant. In this way, more stem cells might survive the treatment and be present to initiate the regenerative process. An alternative, or complementary, approach might be to apply stimulatory factors that shorten the cell cycle following exposure to a cytotoxic agent, on the principle that this might speed up the regenerative process. Clearly, combinations of inhibitors before cytotoxic exposure and stimulators after cytotoxic exposure might in theory represent the most effective approach. It is interesting to note that, if the principles of the experiment are valid, reversal of the sequence, i.e. stimulators before or inhibitors after cytotoxic exposure, might be expected to result not in protection but sensitization of the system.

TGF-βs have been shown to reversibly inhibit the proliferation of epithelial and haematopoietic progenitor cells in mid–late $G_1$ phase (Polyak et al, 1994; Sherr and Roberts, 1995). Maximal inhibition of cell cycling in unsynchronized cells requires that TGF-β be present for one cell cycle period (Geng and Weinberg, 1993). In the prevention of chemotherapy- or radiotherapy-induced oral mucositis, it is critical to optimize the length of time of TGF-β3 exposure, given the evidence that TGF-β3 acts to protect cells by reversible inhibition of cell growth (Sonis et al, 1994). Sufficient preincubation with TGF-β3 is required to take cells out of cycle both before and during exposure to chemotherapy while, ideally, entry of cells back into cycle should occur as soon as the level of chemotherapeutic drug drops below a toxic concentration.

We report here the results of experiments using TGF-β3 administered before a range of doses of gamma rays on the survival of intestinal stem cells. Stem cell function has been measured using the crypt microcolony regeneration assay (Withers and Elkind, 1970; Potten and Hendry, 1985, 1995) over a range of gamma-ray doses and by studying the survival time of animals receiving high doses of radiation. The TGF-β3 was administered over a protracted period of 24 h before the radiation exposure, and this

*Received 30 September 1996*
*Revised 22 November 1996*
*Accepted 29 November 1996*

*Correspondence to*: CS Potten treatment afforded very significant protection both within the microcolony assay and on whole animal survival. The principles of the experimental approach, outlined above, were tested by reversing the sequence (i.e. TGF-β3 after irradiation) in which case sensitization was observed.

### MATERIALS AND METHODS

### Animal studies

BDF1 mice (Paterson Institute), aged 10–12 weeks and weighing about 25 g, housed under conventional conditions with food and water ad libitum and a 12-h light cycle (lights on at 0700 h) were used throughout the experiment. For the crypt microcolony assay, groups of six mice were treated at each dose. Control animals received either radiation alone or saline with 0.1% bovine serum albumin (BSA) (the vehicle for TGF-β3). For the animal survival time studies, groups of 20 mice were used for each treatment protocol. All experimental procedures were conducted in accordance with the recommendations of the Animals (Scientific Procedures) Act 1986, UK.

### Irradiation methods

For the crypt microcolony assay, animals were irradiated in a caesium-137 gamma irradiator at a dose rate of 3.5 Gray (Gy) $min^{-1}$ delivered whole-body to animals breathing pumped air. For the whole animal survival studies, anaesthetized animals were irradiated with X-rays at 300 kVp (HVL 2.3 mm Cu) with the thorax, forelegs and head shielded. The dose rate was 0.62 Gy $min^{-1}$. We have shown that the crypt microcolony survival curves generated using the caesium whole-body irradiation, anaesthetized partial-body X-rays and whole-body X-rays anaesthetized or unanaesthetized were all essentially indistinguishable (Potten, unpublished data). The peak in stem cell DNA synthetic activity in the small intestine is observed at 03.00 h (Potten et al, 1977). Radiation doses were all delivered at the same time of day (03.00 h; making use of a room where the light cycle was reversed so that the radiation could be delivered at 15.00 h real time). The animals were acclimatized to the reverse-cycle room for 2 weeks before an experiment.

### Production, isolation and use of recombinant TGF-β3

Recombinant TGF-β3 was prepared either by purification of conditioned media from CMV/TGF-β3-transfected Chinese hamster ovary (CHO) cells (Stewart et al, 1996) or by the refolding and dimerization of TGF-β3 monomer expressed in *E. coli*, according to the method of Schlunegger et al (1992). Both TGF-β3 preparations demonstrated the same specific activity in vitro ($IC_{50}$ 35 pg $ml^{-1}$; data not shown) using a standard CCL64 growth inhibition assay (Iwata et al, 1985).

The TGF-β3 was dissolved in saline with 0.1% BSA and was delivered intraperitoneally (i.p.) at doses between 0.5 and 10 μg per mouse, per injection, with the majority of the experiments using a dose of 2.5 μg per mouse. A dose of 2.5 μg per mouse is equivalent on a weight basis to 100 μg $kg^{-1}$. The injection protocols generally involved four injections at 24 h, 8 h, 4 h before exposure to ionizing radiation and 0 h with 0 being immediately after irradiation. This represented the standard delivery protocol. Various alternative injection protocols were also investigated (–24, –16, –8, –4 and 0 h), ranging through to protocols in which TGF-β3 was delivered following a dose of radiation. Specifically, other protocol tests were: –32, –24, –8, –4, 0; –24, –8, –4, 0, +4; and +8, +16, +24, +32 h, where + indicates the time after irradiation and – indicates the time before irradiation.

### Crypt microcolony assay

The crypt microcolony assay (Withers and Elkind, 1970) is generally accepted as being a test of the functional capacity of intestinal stem cells to regenerate and maintain the intestinal epithelium. It has been described extensively and used widely (Potten et al, 1983). Briefly, it involves fixation of the entire intestine 3–4 days after radiation and the preparation of routine 3- to 5-μm paraffin sections stained with haematoxylin and eosin, cut transversely to the long axis of the intestine. This will generate cross-sections of the intestine, the perimeter or circumference of each cross-section representing a unit of length within which the number of crypts can be counted. Each crypt is a closed proliferative unit maintained by a number of stem cells that possess the ability to regenerate the crypt by a process of clonal growth and hence have been termed clonogenic cells. A crypt cannot be repopulated by cells from outside the crypt. Each experimental group contained six mice. From each mouse ten cross-sections were obtained and the number of regenerating crypts was counted 3–4 days after irradiation. Thus, for each experimental group, 60 cross-sections were scored. As the probability of observing a regenerating crypt in a section is dependent on the size of the structure, and this may vary between different treatment groups, the size of 15 representative crypts was measured at their widest point and a size correction was applied to the data (Potten et al, 1981). For several of the experiments, a range of irradiation doses was studied and hence survival curves for crypts could be generated. Data for individual mice are presented on the graphs. The DR FIT program (Roberts, 1990) was used with a multitarget model for fitting lines to such curves, for determining the parameters that define the curves with their confidence intervals, and for tests of statistical difference between individual curves using variance-ratio *F*-tests. A significance level of 0.05 was used.

### Survival time experiments

Groups of 20 mice were partial-body irradiated with TGF-β3 administration or saline/BSA before the irradiation according to the standard protocol. The mice were examined four times a day for their health status; any moribund animals were sacrificed and any deaths recorded.

### RESULTS

Using five different radiation doses between 12 and 16 Gy, the number of surviving crypts decreased exponentially with increasing dose, as shown by Figure 1. There was no statistically significant difference between the survival curve obtained with radiation alone and that generated by prior injections of saline/BSA. The $D_0$ value defining the radiation alone curve is 119.3 ± 17.2 cGy and the extrapolation number $N$ is 3115 ± 4784. The $D_0$ is a measure of radiosensitivity and is the reciprocal of the slope of the exponential part of the curve. For the saline/BSA groups, the corresponding figures are $D_0 = 112 \pm 8.1$ cGy and $N = 5093 \pm 4154$.

© Cancer Research Campaign 1997

**Figure 1** Intestinal crypt survival curves for animals irradiated with no other manipulations (●) and animals irradiated with a series of pre-irradiation injections of saline/BSA at –24, –16, –8, –4 and 0 h (■). Each point represents the data from an individual mouse and there were six mice at each dose for each treatment protocol. Lines have been fitted using the Puck formulation in the DR FIT program (Roberts, 1990). No significant difference between the two sets of data was observed ($P = 0.1783$)

**Figure 2** Intestinal crypts survival curves for animals pretreated with saline/BSA (●) with five injections (protocol according to Figure 1) or pretreated with four injections of 2.5 μg of TGF-β3 (■) at –24, –8, –4 and 0 h. A significant difference between the two survival curves was observed ($P < 0.00001$)

As shown in Figure 2, four injections of TGF-β3 administered at –24, –8, –4 h and immediately prior to a dose of radiation results in a survival curve that shows a highly significant shift ($P < 0.00001$) to the right when compared with the curve for saline pretreatment, indicative of increased resistance, i.e. protection of the clonogenic stem cells by the prior treatment with TGF-β3. The parameters that define the TGF-β3 curves are $D_0 = 127.7 \pm 10.6$ cGy and $N = 4639 \pm 3866$. The addition of an extra dose of TGF-β3 16 h before the irradiation, i.e. a protocol involving five doses of TGF-β3 afforded similar levels of stem cell protection (Figure 3). The curve is highly significantly different from the saline/BSA curve ($P < 0.00001$), and the parameters that define the TGF-β3 curve are $D_0 = 146.3 \pm 13.7$ cGy, $N = 11299 \pm 1080$. Figure 4 shows that there is no statistically significant difference between the two modes of administration of TGF-β3 (four doses v five doses).

Table 1 shows the levels of protection afforded by the TGF-β3 at each individual dose, expressed as the ratio (protection factor) of the levels of surviving crypts in the TGF-β3 group compared with the saline/BSA vehicle group. Also shown are examples for which individual doses have been repeated more than once. The effect of increasing TGF-β3 dose per injection on radiation damage induced by 14 Gy radiation is shown in Table 2. An increase in crypt cell protection from 3.3 to 5.4 as the dose of TGF-β3 was increased from 0.5 to 10 μg per injection was observed. Table 3 shows the effect of varying the injection regimens using a standard dose per injection of 2.5 μg. The important observation from this experiment is that TGF-β3 administration after irradiation decreases the protective effect. A single injection 4 h after irradiation with the standard administration before irradiation reduces the protection factor

**Figure 3** Intestinal crypt survival curves for animals pretreated with saline/BSA (five injections, protocol as Figure 1; (●) and five injections of TGF-β3 (2.5 μg; ■). A significant difference between the two curves was observed ($P < 0.00001$)

© Cancer Research Campaign 1997

**Figure 4** Intestinal crypt survival curves comparing the data shown in Figures 2 and 3 for animals pretreated with either four (■) or five (●) injections of 2.5 μg of TGF-β3. The survival curves do not differ significantly from each other ($P = 0.8846$)

from about 4 to 1.6. Administration of TGF-β3 following radiation in fact significantly sensitized the crypts to radiation damage. Here, only a third of the crypts survived compared with the saline/BSA controls.

To effectively reduce these complicating effects of oral and haemopoietic damage, we shielded the head, thorax and forelimbs of the animal in our survival time experiments. Figure 5A shows the survival time for mice receiving 14.5 Gy with and without pretreatment with TGF-β3. It can be seen that TGF-β3 almost completely protects the mice from the effects of this dose of radiation. In the absence of TGF-β3, approximately 65% of the animals will die.

**Table 1** Intestinal crypt cell protection from γ-ray irradiation by prior treatment with TGF-β3

| Dose (Gy) | Protection factor | |
|---|---|---|
| | **Five injections of TGF-β3** | **Four injections of TGF-β3** |
| 12 | 2.94 | 2.94 |
| 13 | 3.15 | 3.33 |
| | 6.00 | 5.88 |
| 14 | 4.87 | 3.84 |
| | | 4.41 |
| 15 | 5.14 | 4.00 |
| 16 | 5.67 | 2.33 |

The protection factor represents the TGF-β3 group mean divided by the saline/BSA group mean. A value of 4.0 means that four times more crypts survived in the TGF-β3-treated group. The five injection groups were administered 2.5 μg TGF-β3 –24, –16, –8, –4, 0 h before irradiation, while the four injection groups were administered 2.5 μg TGF-β3 –24, –8, –4, 0 h before irradiation. Each number represents the ratio from a separate group of six treated and six saline animals.

**Table 2** The effect of TGF-β3 dose on crypt cell survival

| Dose of TGF-β3 (μg) | Protection factor |
|---|---|
| 0.5 | 3.32 |
| 1.5 | 4.05 |
| 2.5 | 4.41 |
| 10 | 5.36 |

The ratios of the crypt survival values obtained in TGF-β3-treated animals compared with those for saline/BSA-treated controls are presented. The dose of TGF-β3 per injection was varied. TGF-β3 was injected at –24, –8, –4 and 0 h before exposure to 14 Gy of gamma rays. The crypt cell protection ratio rose progressively with increasing dose.

Figure 5B shows that once the dose of radiation goes beyond a critical value (here 16 Gy), the levels of damage are such that TGF-β3 can no longer protect the gastrointestinal mucosa. Although more crypts survived (see Figure 3), their numbers are insufficient to maintain animal survival.

**Table 3** Protection of intestinal crypt cells as a function of TGF-β3 administration schedule

| Protocol | Crypts per circumference | Mean ± s.d. | Width (μm) | Size-corrected crypts per circumference | Protection factor |
|---|---|---|---|---|---|
| –24, –8, –4, 0 | 10.1, 17.0, 17.3, 6.4, 28.4, 6.0 | 14.2 ± 7.8 | 37.4 | 11.9 | 3.84 |
| –24, –16, –8, –4, 0 | 17.9, 40.1, 10.9, 10.6 | 19.9 ± 10.6 | 41.2 | 15.1 | 4.87 |
| –32, –24, –8, –4, 0 | 15.9, 14.8, 10.8, 14.2, 11.2, 20.7 | 14.6 ± 3.3 | 42.5 | 10.7 | 3.45 |
| –24, –8, –4, 0, +4 | 3.5, 5.6, 2.6, 7.1, 7.8, 7.0 | 5.6 ± 1.9 | 36.3 | 4.8 | 1.55 |
| +8, +16, +24, +32 | 1.1, 0.7, 1.0, 1.7, 1.1, 1.2 | 1.1 ± 0.3 | 34.6 | 1.0 | 0.32 |
| 14 Gy alone | 0.7, 0.6, 3.5, 1.2, 2.3, 5.3 | 2.4 ± 1.7 | 38.8 | 1.8 | – |
| Untreated animals | 93.6, 107.2, 103.2, 99.4, 97.0, 95.3 | 99.3 ± 4.7 | 31.2 | 99.3 | – |
| Saline/BSA at –24, –16, –8, –4, 0 | 5.8, 2.8, 6.8, 3.6, 3.8, 1.2 | 4.0 ± 1.9 | 40.5 | 3.1 | 1.0 |

TGF-β3 (2.5 μg) was administered by i.p. injection. The '–' symbol denotes time (h) before irradiation (14 Gy in all cases) while the '+' symbol denotes time after irradiation. '0 time' denotes injections delivered immediately after irradiation. Protection factor represents the TFG-β3 group mean divided by the saline/BSA group mean. The individual mean values for the counts of surviving crypts for each mouse are shown together with the width measurements used for the size correction (Potten et al, 1981).

© Cancer Research Campaign 1997

**Figure 5** The survival time of animals exposed to 14.5 Gy (**A**) or 16 Gy (**B**) of 300 kVp X-rays delivered to the abdomen. (**A**) Approximately 65% of the animals pretreated with saline/BSA (–24, –8, –4, 0 h) died between days 3 and 12, while only 5% of the animals pretreated with TGF-β3 succumbed to the effects of radiation. Two animals in the TGF-β3 group died as a consequence of the anaesthesia delivered at the time of irradiation and one animal died between days 6 and 7. The data are presented in terms of percentage survival. (**B**) Animal survival time data following a dose of 16 Gy X-radiation (abdomen only). At this level of radiation dose and damage, administration of TGF-β3 has no effect on animal survival

### DISCUSSION

The transforming growth factor betas are potent inhibitors of cell cycle progression in epithelial and haemopoietic systems. TGF-β3 acts, at least in part, through inhibition of cdk-/cyclin-dependent kinase activity during the $G_1$ phase of the cell cycle (Polyak et al, 1994; Sherr and Roberts 1995). We have recently shown that TGF-β1 administered over a protracted period of time can significantly suppress the proliferative activity in the small intestinal crypts, the effect being particularly pronounced in the stem cell region (Potten et al, 1995). In addition, TGF-βs can have the opposite effect on some cell types and act as indirect mitogens, particularly for mesenchymal cell types, and exert effects on chemotaxis and on the synthesis and degradation of extracellular matrix proteins (Ignotz and Massagué 1986; Posthlethwaite et al, 1987; Wahl et al, 1987; Roberts and Sporn, 1991).

Here, we have shown that the propitious administration of TGF-β3 over a 24-h period before exposure to a cytotoxic agent (radiation) can alter significantly the survival of the crypt stem cells in vivo and increase overall animal survival. It is assumed that this is achieved as a consequence of a reversible TGF-β3-induced arrest of crypt stem cells cycling, thus rendering them more resistant. TGF-β1 or -2 has been shown to reversibly inhibit proliferation of bone marrow stem cells and also to protect mice from the effects of 5-FU over the period of 20–200 days (Grzegorzewski et al, 1994). Our standard reference delivery protocol involved four injections of 2.5 μg per injection per mouse delivered 24 h preceding irradiation (–24, –8, –4, 0 h), with the final injection being delivered immediately after the radiation exposure. Subtle variations of this delivery regimen, by extending the administration time or administering evenly spaced doses of TGF-β3, had little effect on the levels of protection. Increasing the dose per injection had a small beneficial effect. The administration of some of the TGF-β3 injections after exposure to radiation abrogated the positive effect of TGF-β3 pretreatment. Administration of the TGF-β3 following irradiation resulted in a marked sensitization. These data are consistent with the view that TGF-β3 was inhibiting stem cell cycle progression, whereas continued dosing after irradiation would be predicted to inhibit the regenerative process.

The size of the crypts, as determined by their width measurement, suggests that TGF-β3 was affecting the overall cellularity. Pretreatment with TGF-β3 results in crypts 3–4 days after irradiation that have a size slightly greater than observed in animals pretreated with saline/BSA (Table 3). This would be expected if the TGF-β3 enabled extra stem cells to survive per crypt. Continued cell cycle arrest by post-irradiation treatment with TGF-β3, as would be predicted, tended to result in slightly smaller crypts, and this demonstrates the importance of the crypt size correction process (Potten et al, 1981).

There are indications that the stem cells in the crypts show a slightly stronger circadian rhythm than the majority of the crypt cells and have their peak DNA synthetic activity centred around 03.00 h (Potten et al, 1977). It is also believed that the stem cells have a cell cycle duration of approximately 24 h (Potten, 1995). Thus, delivering TGF-β3 over a period of 24 h before a dose of radiation delivered at 03.00 h might be expected to have the maximum effect in preventing cells entering S, i.e. in reducing the number of DNA synthesizing cells in the stem cell compartment at the time of irradiation. It remains to be seen whether other protocols with different spacings between the TGF-β3 injections might be more effective. The current experiments, in which the radiation was delivered at 03.00 h, resulted in better levels of protection than were observed in earlier preliminary experiments in which the time of irradiation tended to be between 09.00 and 12.00 h.

The well-being of the animal, and similarly of a patient undergoing cancer therapy treatment, will depend on the competitive interaction between cellular depletion processes and cellular regeneration processes in the gastrointestinal tract. If the former are too severe, or the latter too slow, the consequence is loss of mucosal integrity, which ultimately results in the symptoms of the gastrointestinal radiation syndrome. If these symptoms are too severe, the animal will die from gastrointestinal damage, and this characteristically occurs over the period of about days 4–12. The survival of intestinal crypts is determined by the level of

© Cancer Research Campaign 1997

survival of individual clonogenic stem cells within each crypt. The survival and well-being of the animal depends ultimately on the levels of surviving crypts (Hendry et al, 1983).

Following whole-body irradiation, survival of animals is influenced in a complicated fashion by damage induced in the oral mucosae and in the haemopoietic system. The latter is inherently more sensitive than the gastrointestinal tract. Deaths attributed to bone marrow damage usually occur later than 12 days. In order to reduce the consequences of this oral and haemopoietic damage, we chose to irradiate only the abdomens of the animals in the survival study. The survival time experiment was used as a paradigm for patient well-being in a cancer treatment situation, and these experiments can be viewed independently of the crypt survival studies. However, the survival curves shown in Figures 2 and 3 show that TGF-β3 can have a fairly dramatic effect on the fraction of crypts surviving. The TGF-β3 treatment effectively prevents damage equivalent to 2 Gy of radiation, and the consequences of this can be seen very dramatically in the survival time study illustrated in Figure 5A – in the absence of TGF-β3 65% of the animals die after 14.5 Gy, while in the TGF-β3-treated group only 5% die. If the dose is raised to 16 Gy, only about 0.5% of the crypts survive in the absence of TGF-β3 (Figure 1). TGF-β3, may in principle, protect some clonogenic cells and raise this to about 2.5%, but this is still at a level of damage that results in animal death (Figure 5B).

The principles of these experiments may have major clinical implications in terms of their potential for improving the quality of life of cancer therapy patients. Furthermore, they may possibly offer the opportunity of dose escalation and, hence, improvements in cure rate. However, there are many questions still to be resolved. We have chosen to use radiation as a model cytotoxic for which the dose and dose distribution can be fairly precisely controlled. We need to demonstrate that TGF-β3 can protect the intestinal epithelium against other forms of cytotoxic agents. Cancer chemotherapy involves the use of multiple agents delivered repeatedly over a protracted period. There are no adequate animal models for such combination therapies. We are currently developing such models and the efficacy of TGF-β3 treatment within this context needs to be tested. Finally, there is the question of to what extent cells in tumours may be protected by TGF-β3 along with the normal tissue. Although this is not fully resolved, there is strong evidence indicating that tumour cells are less responsive to the cell cycle progression inhibition of TGF-β3 by abrogation of the signalling pathways, leading to the phosphorylation of the retinoblastoma (Rb) gene product (Sherr and Roberts 1995).

### ACKNOWLEDGEMENTS

This work has been supported by the Cancer Research Campaign (UK) and by Oncogene Science (Uniondale, NY, USA). We are grateful to the staff of Laboratory 19 and the histology laboratory for their continued valuable assistance. Many members of the Department of Epithelial Biology helped with these experiments for which we are extremely grateful. We thank Arthur Bruskin for helpful suggestions and comments during the course of these studies.

### REFERENCES

Chabner BA (1993) Anticancer drugs In *Cancer, Principles and Practice of Oncology*, 4th edn, DeVita VT, Hellman S and Rosenberg SA. (eds), pp. 325–417, Lipincott: Philadelphia

Geng Y and Weinberg RA (1993) Transforming growth factor β effects on expression of G1 cyclins and cyclin-dependent kinases. *Proc Natl Acad Sci* **90**: 10315–10319

Grzegorzewski K, Ruscetti FW, Usui N, Damia G, Longo DL, Carlino HA, Keller JR and Wiltrout RH (1994) Recombinant Transforming Growth Factory $\beta_1$ and $\beta_2$ protected mice from acutely lethal doses of 5-Fluorouracil and doxorubicin. *J Exp Medicine* **180**: 1047–1057

Hendry JH, Potten CS and Roberts NP (1983) The gastrointestinal syndrome and mucosal clonogenic cells relationship between target cell sensitivities, $LD_{50}$ and cell survival, and their modification by antibiotics. *Radiat Res* **96**: 100–112

Ignotz RA and Massagué J (1986) Transforming growth factors β stimulate the expression of fibronectin and collagen and their incorporation into extra cellular matrix. *J Biol Chem* **261**: 4337–4345

Iwata KK, Fryling CM, Knott WB and Todaro GJ (1985) Isolation of tumour cell growth inhibiting factors from human rhabdomyosarcoma cell line. *Cancer Res* **45**: 2689–2694

Morstyn G, Foote M, Perkins D and Vincent D (1994) The clinical utility of granulocyte colony stimulating factor: early achievements and future promise. *Stem Cells* (suppl. 1): 213–228

Polyak K, Kato J-Y, Solomon MJ, Sherr CJ, Massagué J, Roberts JM and Koff A (1994) p27kipl, a cyclin-Cdk inhibitor, links transforming growth factor-β and contact inhibition to cell cycle arrest. *Genes Devel* **8**: 9–22

Posthlethwaite AE, Keski-Oja J, Moses HL and Kang AH (1987) Migration of human fibroblasts by transforming growth factor Beta. *J Exp Med* **65**: 251–256

Potten CS (1995) Structure, function and proliferative organisation of mammalian gut. In *Radiation and Gut*, Potten CS and Hendry JH. (eds), pp. 1–31. Elsevier Science: Amsterdam

Potten CS and Hendry JH (1985) The microcolony assay in mouse small intestine. In *Cell Clones: Manual of Mammalian Cell Techniques*, Potten CS and Hendry JH. (eds), pp. 50–60. Churchill Livingstone: Edinburgh

Potten CS and Hendry JH (1995) Clonal regeneration studies. In *Radiation and Gut*, Potten CS and Hendry JH (eds), pp. 45–99. Elsevier: Amsterdam

Potten CS, Al-Barwari SE, Hume WJ and Searle J (1977) Circadian rhythms of presumptive stem cells in three different epithelia of the mouse. *Cell Tissue Kinet* **10**: 557–568

Potten CS, Rezvani M, Hendry JH, Moore JV and Major D (1981) The correction of intestinal microcolony counts for variations in size. *Int J Radiat Biol* **40**: 321–326

Potten CS, Hendry JH, Moore JV and Chwalinski S (1983) Cytotoxic effects in gastrointestinal epithelium (as exemplified by small intestine). In *Cytotoxic Insult to Tissue*, Potten CS and Hendry JH. (eds), pp. 105–152. Churchill Livingstone: Edinburgh

Potten CS, Owen G, Hewitt D, Chadwick CA, Hendry JH, Lord BI and Woolford LB (1995) Stimulation and inhibition of proliferation in the small intestinal crypts of the mouse after in vivo administration of growth factors. *Gut* **36**: 864–873

Roberts SA (1990) Drfit: a program for fitting radiation survival models. *Int J Radiat Biol* **57**: 1234–1246

Roberts AB and Sporn MB (1991) The transforming growth factor -βs. In *Peptide Growth Factors and their Receptors* I Sporn MB and Roberts AB. (eds), pp. 419–472. Springer-Verlag: New York

Schlunegger MP, Cerletti N, Cox DA, McMaster GK, Schmitz A and Grutter MG (1992) Crystallization and preliminary x-ray analysis of recombinant human transforming growth factor beta-2. *FEBS Lett* **303**: 91–93

Sherr CJ and Roberts JM (1995) Inhibitors of mammalian GI cyclin-dependent kinases. *Genes Devel* **9**: 1149–1163

Sonis S, Lindquist L, Van Vugt A, Stewart A, Stam K, Qu G-Y, Iwata K and Haley JD (1994) Prevention of chemotherapy-induced ulcerative mucositis by transforming growth factor β3. *Cancer Res* **54**: 1135–1138

Stewart AA, Haley JD, Qu G-Y, Stam K, Fenyon D, Chait BT, Marshak DR and Iwata K (1996) Isolation and physical characterization of native and recombinant transforming growth factor beta-3. *Growth Factor* **13**: 1–11

Wahl SM, Hunt DA, Wakefield LM, McCartney-Francis N, Wahl L, Roberts AB and Sporn MB (1987) Transforming growth factor beta (TGFβ3) indices monocyte chemotoxis and growth factor production. *Proc Natl Acad Sci USA* **84**: 5788–5792

Withers HR and Elkind MM (1970) Micro-colony survival assay for cells of mouse intestinal mucosa exposed to radiation. *Int J Radiat Biol* **7**: 261–268

© Cancer Research Campaign 1997


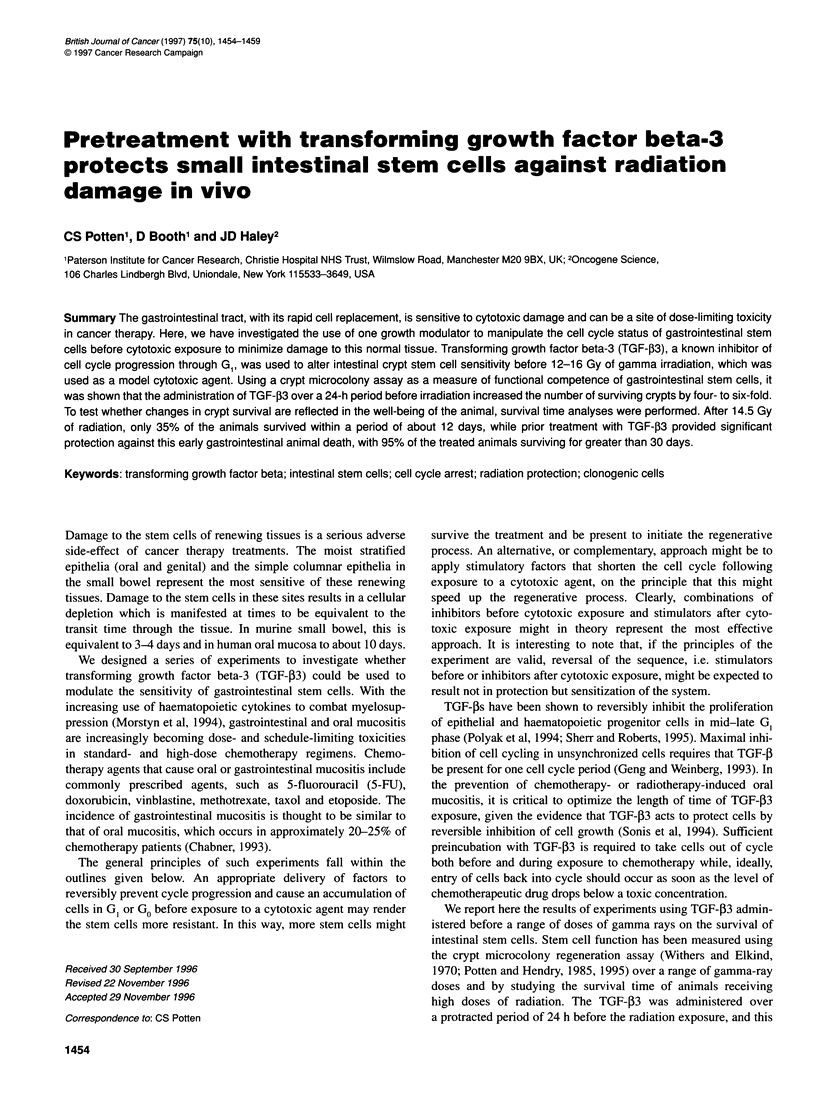

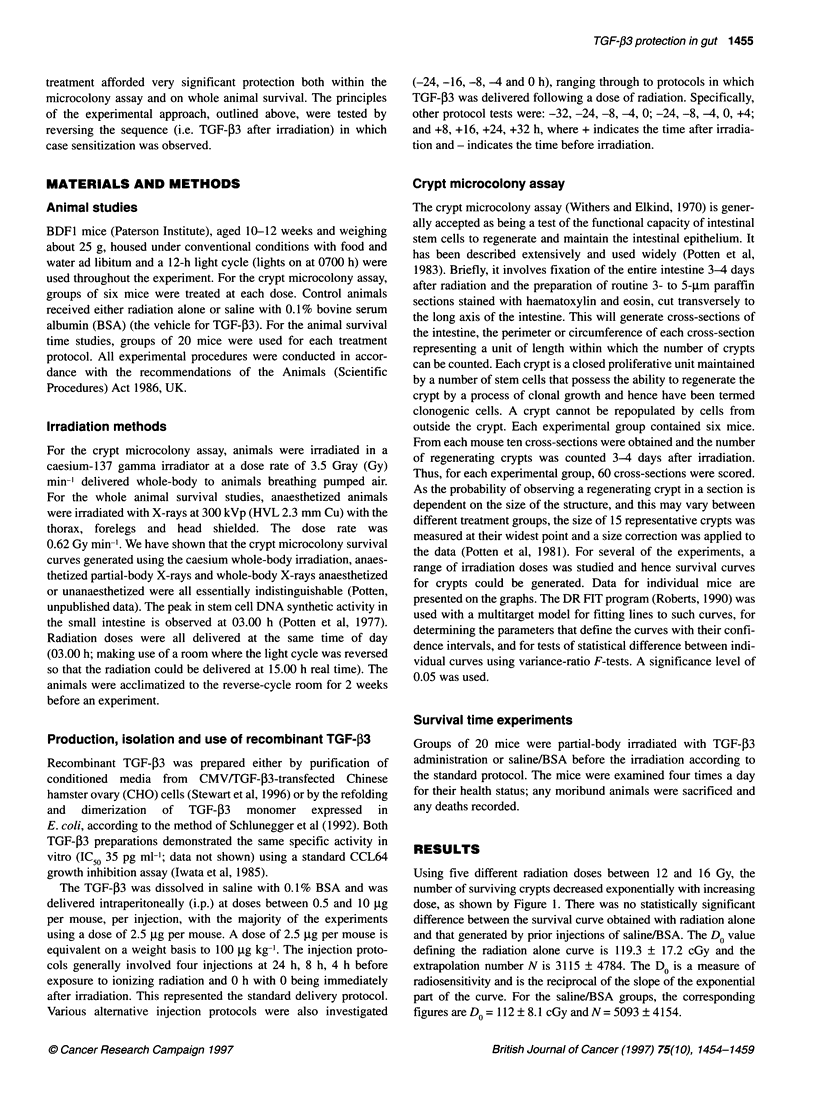

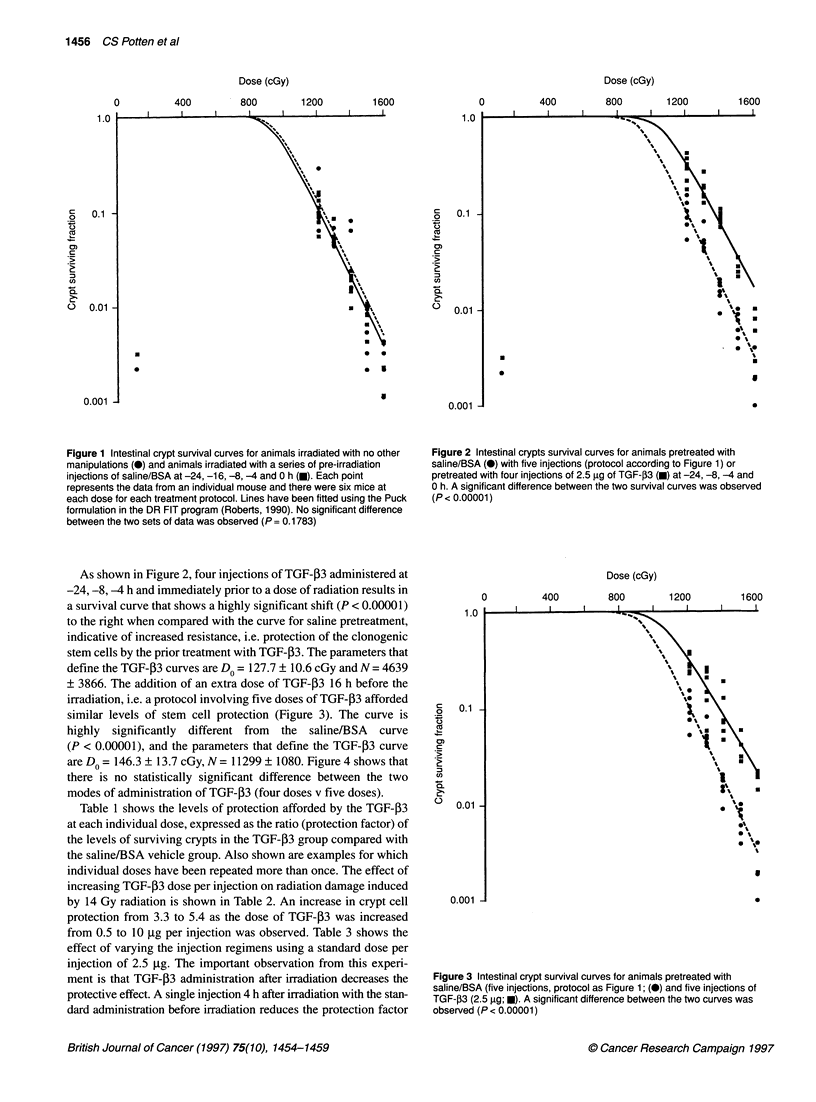

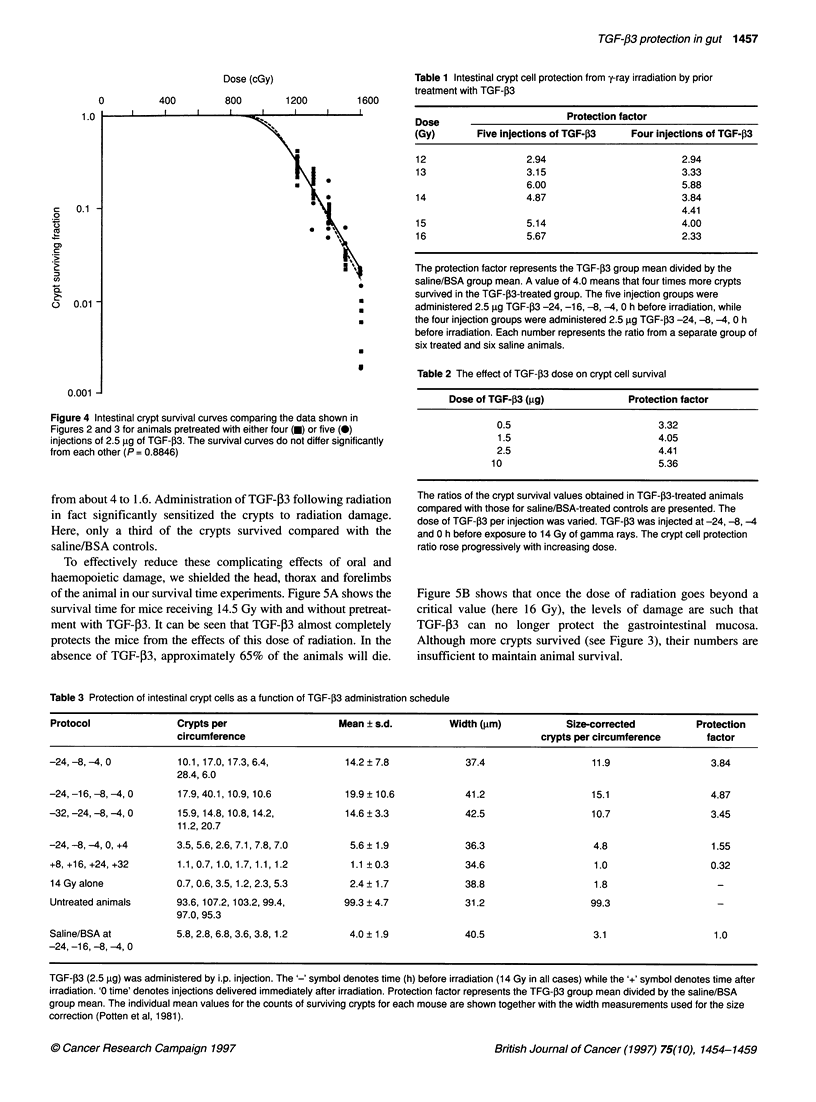

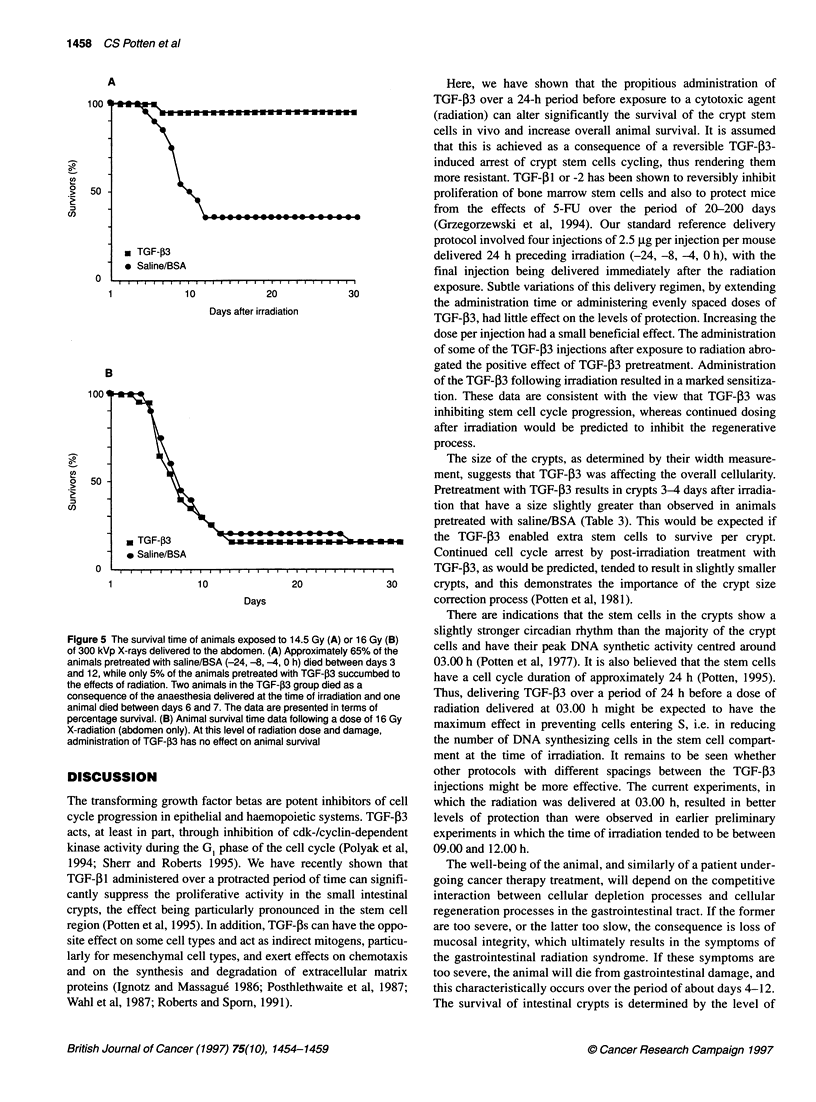

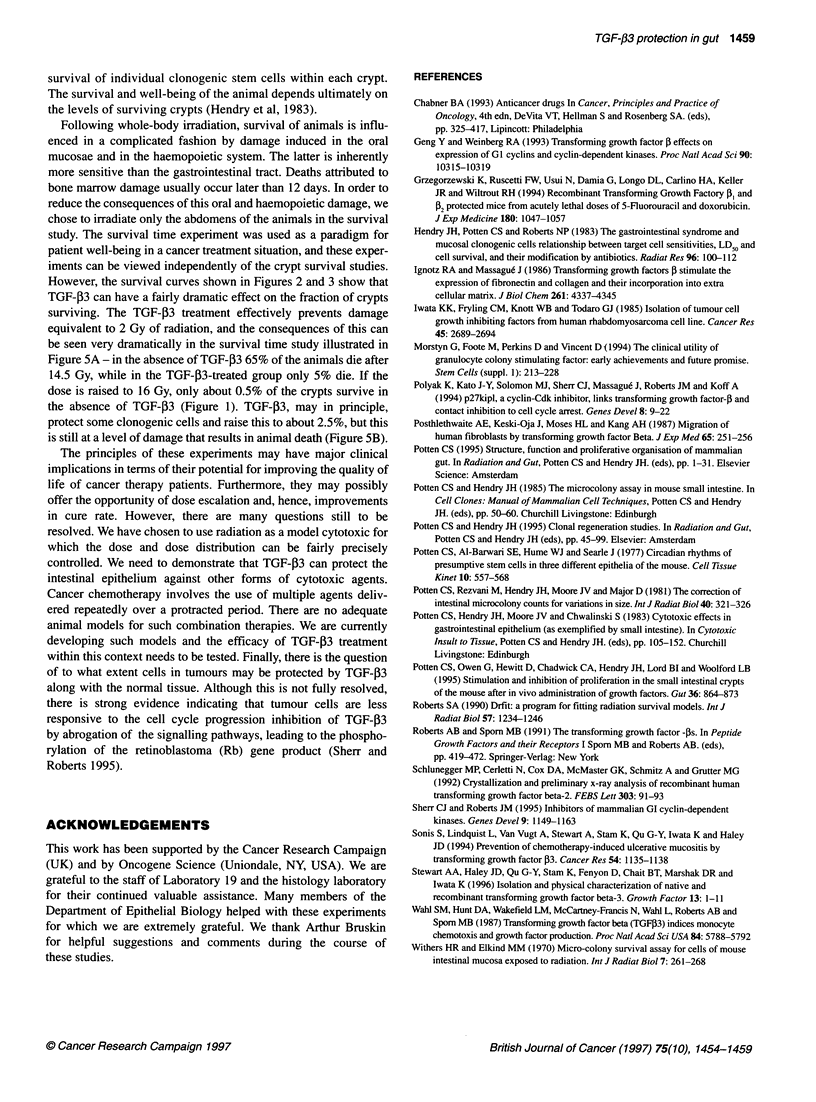


## References

[OCR_00735] Geng Y., Weinberg R. A. (1993). Transforming growth factor beta effects on expression of G1 cyclins and cyclin-dependent protein kinases.. Proc Natl Acad Sci U S A.

[OCR_00740] Grzegorzewski K., Ruscetti F. W., Usui N., Damia G., Longo D. L., Carlino J. A., Keller J. R., Wiltrout R. H. (1994). Recombinant transforming growth factor beta 1 and beta 2 protect mice from acutely lethal doses of 5-fluorouracil and doxorubicin.. J Exp Med.

[OCR_00746] Hendry J. H., Potten C. S., Roberts N. P. (1983). The gastrointestinal syndrome and mucosal clonogenic cells: relationships between target cell sensitivities, LD50 and cell survival, and their modification by antibiotics.. Radiat Res.

[OCR_00750] Ignotz R. A., Massagué J. (1986). Transforming growth factor-beta stimulates the expression of fibronectin and collagen and their incorporation into the extracellular matrix.. J Biol Chem.

[OCR_00755] Iwata K. K., Fryling C. M., Knott W. B., Todaro G. J. (1985). Isolation of tumor cell growth-inhibiting factors from a human rhabdomyosarcoma cell line.. Cancer Res.

[OCR_00760] Morstyn G., Foote M., Perkins D., Vincent M. (1994). The clinical utility of granulocyte colony-stimulating factor: early achievements and future promise.. Stem Cells.

[OCR_00765] Polyak K., Kato J. Y., Solomon M. J., Sherr C. J., Massague J., Roberts J. M., Koff A. (1994). p27Kip1, a cyclin-Cdk inhibitor, links transforming growth factor-beta and contact inhibition to cell cycle arrest.. Genes Dev.

[OCR_00770] Postlethwaite A. E., Keski-Oja J., Moses H. L., Kang A. H. (1987). Stimulation of the chemotactic migration of human fibroblasts by transforming growth factor beta.. J Exp Med.

[OCR_00787] Potten C. S., Al-Barwari S. E., Hume W. J., Searle J. (1977). Circadian rhythms of presumptive stem cells in three different epithelia of the mouse.. Cell Tissue Kinet.

[OCR_00801] Potten C. S., Owen G., Hewitt D., Chadwick C. A., Hendry H., Lord B. I., Woolford L. B. (1995). Stimulation and inhibition of proliferation in the small intestinal crypts of the mouse after in vivo administration of growth factors.. Gut.

[OCR_00792] Potten C. S., Rezvani M., Hendry J. H., Moore J. V., Major D. (1981). The correction of intestinal microcolony counts for variation in size.. Int J Radiat Biol Relat Stud Phys Chem Med.

[OCR_00805] Roberts S. A. (1990). DRFIT: a program for fitting radiation survival models.. Int J Radiat Biol.

[OCR_00814] Schlunegger M. P., Cerletti N., Cox D. A., McMaster G. K., Schmitz A., Grütter M. G. (1992). Crystallization and preliminary X-ray analysis of recombinant human transforming growth factor beta 2.. FEBS Lett.

[OCR_00819] Sherr C. J., Roberts J. M. (1995). Inhibitors of mammalian G1 cyclin-dependent kinases.. Genes Dev.

[OCR_00823] Sonis S. T., Lindquist L., Van Vugt A., Stewart A. A., Stam K., Qu G. Y., Iwata K. K., Haley J. D. (1994). Prevention of chemotherapy-induced ulcerative mucositis by transforming growth factor beta 3.. Cancer Res.

[OCR_00834] Wahl S. M., Hunt D. A., Wakefield L. M., McCartney-Francis N., Wahl L. M., Roberts A. B., Sporn M. B. (1987). Transforming growth factor type beta induces monocyte chemotaxis and growth factor production.. Proc Natl Acad Sci U S A.

[OCR_00839] Withers H. R., Elkind M. M. (1970). Microcolony survival assay for cells of mouse intestinal mucosa exposed to radiation.. Int J Radiat Biol Relat Stud Phys Chem Med.

